# Evaluation of Serum Apolipoprotein A1 in Canine Sepsis

**DOI:** 10.3389/fvets.2020.00263

**Published:** 2020-05-13

**Authors:** Massimo Giunti, Giorgio Grossi, Roberta Troía, Federico Fracassi, Francesco Dondi

**Affiliations:** Department of Veterinary Medical Sciences, Alma Mater Studiorum, University of Bologna, Bologna, Italy

**Keywords:** high-density lipoproteins, acute phase response, dogs, prognosis, septic peritonitis

## Abstract

Decreased serum apolipoprotein A1 (Apo-A1) concentration is associated with mortality in human sepsis. The diagnostic and prognostic role of serum Apo-A1 concentrations in canine sepsis was evaluated. Serum samples from septic dogs (*n* = 91) and healthy controls (*n* = 15) were retrospectively analyzed. According to the sepsis origin, four categories were identified: parvoviral enteritis (*n* = 26), pyometra (*n* = 20), septic peritonitis (*n* = 19), and miscellanea (*n* = 26). The canine acute patient physiologic and laboratory evaluation fast score (APPLE_fast_), serum C-reactive protein (CRP) and albumin concentrations were reviewed in all enrolled dogs. Increased CRP (252.6 ± 119.2 mg/L; Reference Interval: 0–8.5 mg/L) and significant lower serum albumin and Apo-A1 concentrations were documented in dogs with sepsis (22.8 ± 5.3 g/L and 1.17 ± 0.27 g/L, respectively) compared to healthy ones (33.1 ± 2.5 g/L and 1.32 ± 0.05 g/L, respectively) (*P* < 0.0001). According to the origin of sepsis, only the subgroup of dogs with septic peritonitis had significantly lower Apo-A1 (1.03 ± 0.26 g/L) concentrations compared to healthy dogs (*P* < 0.001). No significant differences were found in serum albumin and CRP concentrations, and in APPLE_fast_ score values among the different subgroups of sepsis. Diagnosis of septic peritonitis was associated with a higher frequency of death (*P* = 0.006). In septic dogs, significant lower Apo-A1 concentrations were detected in non-survivors (1.02 ± 0.28 g/L; *n* = 27) compared to survivors (1.23 ± 0.24 g/L; *n* = 64; *P* = 0.0007). Moreover, significant higher values of the APPLE_fast_ score were calculated in non-survivors (26 ± 4; *n* = 19) compared to survivors (23 ± 4; *n* = 51) (*P* = 0.0114). According to the area under the ROC curve analysis, Apo-A1 <96 mg/dl had a fair accuracy (AUC = 0.72) to correctly predict mortality (*P* = 0.0004). Apo-A1 might support a diagnosis of canine septic peritonitis with a potential prognostic significance. Further prospective studies are warranted.

## Introduction

Apolipoprotein A1 (Apo-A1) is a major protein component of high-density lipoproteins (HDL) and a key determinant of HDL formation. HDL, which play a major role in reverse cholesterol transport, may also be involved in modulating the inflammatory response during sepsis. Significant anti-inflammatory effects of HDL have been demonstrated both *in vitro* ad *in vivo*, possibly as a result of: lipopolysaccharide (LPS) binding and neutralization; inhibition of adhesion molecule expression; stimulation of endothelial nitric oxide synthase production; and antioxidant activity (1). During sepsis, lower serum LPS neutralizing activity, less LPS clearance, impaired leukocyte recruitment, and reduced corticosterone generation were reported in Apo-A1 knockout mice; moreover, they showed a moderately decreased survival in Gram-positive bacterial infection compared with control mice (2). In human patients with severe clinical signs related to sepsis, HDL are shifted to acute phase HDL, which are enriched in serum amyloid A (SAA) and depleted of cholesterol and Apo-A1 (3). Specifically, decreased serum Apo-A1 concentration is associated with greater illness severity, lethality, and susceptibility to infection in critical patients (4). Apo-A1 has been recently measured in a group of dogs with leishmaniosis, which had significantly decreased serum concentrations of this protein compared to healthy controls; furthermore, dogs with a good response to anti-Leishmania treatment displayed a significant increase of Apo-A1, suggesting its role as a potential biomarker of treatment monitoring during this disease (5). Curiously, Milanović et al. reported a parallel Apo-A1 and SAA serum concentration increase in a population of dogs with acute infection by *Babesia canis* compared to control dogs; however, the latter finding is still unexplained and warrants further assessment (6).

To our knowledge, no information is available on the prognostic significance of circulating Apo-A1 during canine sepsis. The present study aimed to evaluate the diagnostic and prognostic role of serum Apo-A1 concentrations in hospitalized dogs with sepsis. We hypothesized that serum concentrations of Apo-A1 are reduced in septic dogs and are able to predict outcome in terms of survival at hospital discharge.

## Materials and Methods

This study involved a retrospective analysis on stored samples collected in a population of dogs diagnosed with sepsis and prospectively enrolled in a previous study performed at a Veterinary University Hospital from February 2015 to October 2017. The study was approved by the local Institutional Animal Care and Use Committee.

### Animals

Dogs were diagnosed with sepsis if they exhibited two or more of the following criteria for systemic inflammatory response syndrome (SIRS): body temperature <38.1°C or >39.2°C; heart rate >120/min; respiratory rate >20/min; white blood cell count <6,000/μL or >16,000/μL or percentage of band neutrophils >3% of the total white blood cell count (7), and if an infection was confirmed by cytology, microbiology, histopathology or real-time polymerase chain reaction. To be included in the present study, in order to guarantee an adequate storage stability during the study period (8, 9), an aliquot of serum sample, immediately processed after collection and stored frozen (−80°C), had to be available for Apo-A1 measurement. Patients with diseases or conditions able to influence Apo-A1 concentration such as chronic liver disease, hyperlipidemia, or receiving total parenteral nutrition (10) at the time of blood sampling were excluded from the study. Serum samples from dogs, defined healthy according to history, physical examination, complete blood count (CBC), and serum chemistry, were also available (*n* = 15).

### Data Collection

Attending ICU clinicians were responsible for the clinical management of the patients included in the study. At the time of presentation at our VUH, the following data were recorded: signalment and history, clinical findings including rectal temperature, heart rate, respiratory rate, and mental status. The canine acute patient physiologic and laboratory evaluation fast score (APPLE_fast_) was calculated at the time of hospital admission in order to assess disease severity, as previously described (11). Dogs were classified according to their outcome as survivors (survived to hospital discharge) or non-survivors (died despite medical treatment or humanely euthanized by the clinical investigators due to moribund conditions or end-stage disease) and the length of hospital stay was recorded and included as a variable under analysis. Dogs that were euthanized for financial reasons were excluded from the study. Hematology and chemistry profiles, including serum C-reactive protein (CRP) and albumin concentrations, obtained upon hospital admission and coincident with the collection time of samples for Apo-A1 analysis, were reviewed in all enrolled septic dogs. CBC was determined by an automated blood cell counter (ADVIA 2120 Hematology System, Siemens Healthcare Diagnostics, USA). CRP was measured by using an immunoturbidimetric assay that had been previously validated by our group for canine serum samples (CRP OSR6147, Olympus/Beckman Coulter, O'Callaghan's Mills, Ireland). Apo-A1 immunoassay was set up using calibration (Apo-A1 and B calibrator; Olympus/Beckman Coulter, O'Callaghan's Mills, Ireland) and quality control (Control serum L1 and L2; Olympus/Beckman Coulter, O'Callaghan's Mills, Ireland) material provided by the manufacturer. The assay was validated in our lab for dogs following a standard validation protocol including linearity and spiking recovery, and precision. Linearity was evaluated in triplicate diluting a canine pooled sample with a concentration of 304 mg/dL of Apo-A1. Regression equation was the following: *y* = 1.672 + 0.331 (*r* = 1.00; *P* < 0.001). Spiking recovery results were 95 and 102% using a sample with “normal” (215 mg/dL) and “low” (45 mg/dL) expected concentration of Apo-A1, respectively. Four pooled samples at various concentration (240, 120, 60, and 30 mg/dL) of Apo-A1 were used to evaluate inter- and intra-assay coefficient of variation (CV). Mean intra-assay CV (ten replicate of samples evaluated in triplicate in the same working day) was 2.2%. Mean inter-assay CV (five replicate in 5 following days evaluated in triplicate) was 1.5%.

### Statistical Analysis

Data distribution was assessed graphically and using the D'Agostino Pearson test. The independent samples *t*-test was used to compare the means of continuous variables between groups. To compare the equality of the variances, the *F*-test was performed. If the *P*-value was low (*P* < 0.05) a correction for unequal variances was performed by the Welch test. One-way analysis of variance was used to test the difference between the means of subgroups of septic dogs. *Post-hoc* test was performed by the Tukey-Kramer test. Receiver operating characteristic (ROC) curve analysis was used to find optimal cut-off values for variables predicting prognosis and to calculate the area under the ROC curve (AUC). The Chi-squared test was performed to evaluate the statistical significance of differences between categorical variables. Correlations between continuous variables were determined using the Pearson correlation coefficient. A value of *P* < 0.05 was considered significant. Statistical analysis was performed using a statistical software package [MedCalc Statistical Software version 19.1.3 (MedCalc Software bv, Ostend, Belgium; https://www.medcalc.org; 2019)].

## Results

Serum samples from 91 septic dogs were available for Apo-A1 analysis. Therefore, it was possible to identify four subgroups according to the sepsis origin: parvoviral enteritis (*n* = 26), pyometra (*n* = 20), septic peritonitis (*n* = 19), and miscellanea (*n* = 26). The septic diseases included in the miscellanea group were the following: necrotizing fasciitis (*n* = 9), pneumonia (*n* = 3), prostatitis/urinary tract infection (*n* = 3), pyothorax (*n* = 3), bacterial cholangitis (*n* = 2), pyoderma (*n* = 2), bite wound infection (*n* = 1), renal abscess (*n* = 1), perineal abscess (*n* = 1), blood stream infection (*n* = 1). Median age was 7 years (range 0.1–17.5) and median body weight was 17.2 kg (range 0.6–55.7). Thirty-seven breeds were represented with the most common being mixed breed (*n* = 33), Labrador Retriever (*n* = 6), German Shepherd (*n* = 5), French Bulldog (*n* = 3), American Staffordshire Terrier (*n* = 3). Demographic information in each subgroup of sepsis and in healthy dogs are reported in Table 1. The breeds represented in the latter group were: mixed breed (*n* = 10), American Staffordshire Terrier (*n* = 1), Australian Shepherd (*n* = 1), Boxer (*n* = 1), Bullmastiff (*n* = 1), Labrador Retriever (*n* = 1).

**Table 1:** Demographic data in the subgroups of septic dogs (*n* = 91) and in healthy dogs (*n* = 15).

	**Septic peritonitis (*n* = 19)**	**Pyometra (*n* = 20)**	**Parvoviral enteritis (*n* = 26)**	**Miscellanea (*n* = 26)**	**Healthy dogs (*n* = 15)**	***P*-value**
Age (months)	96 (18–198)^*^	120 (12–180)^*^	4 (1–12)	120 (4.8–210)^*^^¥^	36 (12–72)	<0.0001
Body weight (Kg)	25.7 (3.5–55.7)^*^	21.6 (2–44.8)	7.9 (0.6–27.6)	22 (6–36)^*^	24.5 (6–63)^*^	<0.001
Intact females	4	20	9	2	4	ND
Spayed females	5	–	1	3	2	ND
Intact males	7	–	16	17	6	ND
Castrated males	3	–	–	2	3	ND

*Results are presented as median and minimum and maximum value (age; body weight) or total number of dogs; P < 0.05 indicates a significant difference; ND, not determined*;

* *Significant difference from parvoviral enteritis*;

¥ *Significant difference from healthy dogs*.

**Figure 1:**
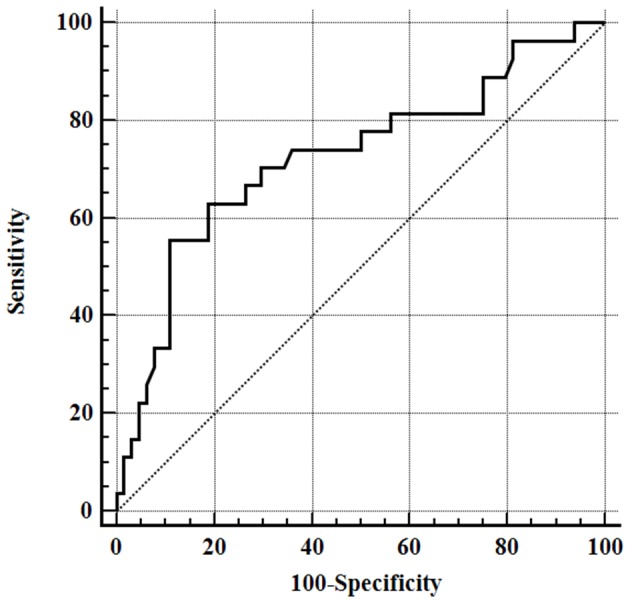
Receiver operating characteristic (ROC) curve for outcome prediction of apolipoprotein-A1 serum concentrations obtained from all septic dogs, area under the ROC curve (AUC) 0.725 (95% CI = 0.621–0.813), *P* = 0.0004.

Results for serum albumin concentration and APPLE_fast_ score were available for 74 and 70 septic dogs, respectively. Mean serum albumin and Apo-A1 concentrations were significantly lower in dogs with sepsis compared to healthy ones, while mean serum CRP concentrations were above the reference interval in the entire population of septic dogs (Table 2). When comparisons were made according to the origin of sepsis, only the subgroup of dogs with septic peritonitis had significantly lower Apo-A1 concentrations compared to healthy dogs (Table 3). Furthermore, both dogs with septic peritonitis and parvoviral enteritis had significantly lower Apo-A1 concentrations compared to dogs with pyometra (Table 3). On the other hand, no significant differences were found in serum albumin and CRP concentrations, and in APPLE_fast_ score values among the different subgroups of sepsis (Table 3). Sixty-four out of 91 dogs (70%) survived to hospital discharge, while 27/91 (30%) died or were humanely euthanized due to the severe prognosis. Diagnosis of septic peritonitis was significantly associated with a higher frequency of death compared to the other causes of sepsis in this population (*P* = 0.006). In the whole septic population, significantly lower serum Apo-A1 concentrations were detected in non-survivors compared to survivors; moreover, non-survivors dogs had significantly higher values of the APPLE_fast_ score compared to survivors (Table 4). According to the ROC curve analysis, a serum Apo-A1 <0.96 g/L had a 55.6% sensitivity and a 89.1% specificity (AUC = 0.725) to correctly predict mortality (*P* = 0.0004; Figure 1). In dogs surviving to hospital discharge, baseline values of Apo-A1 had a significant negative correlation with the length of stay in hospital (*r* = −0.33; *P* = 0.017).

**Table 2:** Results of selected variables (albumin, apolipoprotein A1, C-reactive protein, APPLE_fast_ score) in septic (*n* = 91) and in healthy dogs (*n* = 15).

	**Septic dogs (*n* = 91)**	**Healthy dogs (*n* = 15)**	**R.I**.	***P*-value**
Albumin (g/L)	22.8 ± 5.3 (*n* = 74)	33.1 ± 2.5 (*n* = 15)	27.5–38.5	<0.0001
Apo-A1 (g/L)	1.17 ± 0.27 (*n* = 91)	1.32 ± 0.05 (*n* = 15)	ND	<0.0001
CRP (mg/L)	252.6 ± 119.2 (*n* = 91)	ND	0–8.5	ND
APPLE_fast_ score	24 ± 4 (*n* = 70)	ND	ND	ND

*Results are presented as mean ± standard deviation. APPLE_fast_, canine acute patient physiologic and laboratory evaluation; ND, not determined; R.I., reference interval; Apo-A1, Apolipoprotein A1; CRP, C-reactive protein; P < 0.05 indicates a significant difference*.

**Table 3:** Results of selected variables (albumin, apolipoprotein A1, C-reactive protein, APPLE_fast_ score) in the subgroups of septic dogs (*n* = 91) and in healthy dogs (*n* = 15).

	**Septic peritonitis**	**Pyometra**	**Parvoviral enteritis**	**Miscellanea**	**Healthy dogs**	***P*-value**
Albumin (g/L)	21.3 ± 6.2 (*n* = 18)^*^	25.0 ± 5.6 (*n* = 17)^*^	22.1 ± 5.0 (*n* = 18)^*^	22.9 ± 4.2 (*n* = 21)^*^	33.1 ± 2.5 (*n* = 15)	<0.001
Apo-A1 (g/L)	1.03 ± 0.26 (*n* = 19)^*^^¥^	1.35 ± 0.21 (*n* = 20)	1.13 ± 0.28 (*n* = 26) ^¥^	1.18 ± 0.26 (*n* = 26)	1.32 ± 0.05 (*n* = 15)	<0.001
CRP (mg/L)	259.7 ± 123.3 (*n* = 19)	279.5 ± 115 (*n* = 20)	217.2 ± 94.3 (*n* = 26)	272.4 ± 139 (*n* = 26)	ND	0.346
APPLE_fast_ score	24 ± 4 (*n* = 18)	23 ± 4 (*n* = 16)	23 ± 4 (*n* = 16)	25 ± 5 (*n* = 20)	ND	0.256

*Results are presented as mean ± standard deviation. APPLE_fast_, canine acute patient physiologic and laboratory evaluation; ND, not determined; P < 0.05 indicates a significant difference*;

* *Significant difference from healthy dogs; Apo-A1, Apolipoprotein A1; CRP, C-reactive protein*;

¥ *Significant difference from pyometra*.

**Table 4:** Results of selected variables (albumin, apolipoprotein A1, C-reactive protein, APPLE_fast_ score, days of hospitalization) in septic dogs classified as survivors (*n* = 64) and non-survivors (*n* = 27).

	**Survivors**	**Non survivors**	**R.I**.	***P*-value**
Albumin (g/L)	23.3 ± 5.1 (*n* = 52)	21.5 ± 5.8 (*n* = 22)	27.5–38.5	0.187
Apo-A1 (g/L)	1.23 ± 0.24 (*n* = 64)	1.02 ± 0.28 (*n* = 27)	ND	0.0003
CRP (mg/L)	258.4 ± 129.7 (*n* = 64)	238.8 ± 90.1 (*n* = 27)	0–8.5	0.410
APPLE_fast_ score	23 ± 4 (*n* = 51)	26 ± 4 (*n* = 19)	ND	0.0114
Days of hospitalization	5.5 (2–20)	2.5 (1–12)	ND	0.0095

*Results are presented as mean ± standard deviation. Days of hospitalization are reported as median and minimum and maximum value. APPLE_fast_, Canine Acute Patient Physiologic and Laboratory Evaluation; ND, not determined; Apo-A1, Apolipoprotein A1; CRP, C-reactive protein; P < 0.05 indicates a significant difference*.

## Discussion

Acute inflammatory diseases in humans, including infections and sepsis, determine a reduction of circulating levels of Apo-A1 and high-density lipoprotein cholesterol, and affect HDL composition. Altogether, these changes result in the loss of HDL functional properties, including protection against sepsis (12). Low plasma concentrations of high-density lipoprotein cholesterol inversely correlate with the severity of the septic disease and associate with an exaggerated systemic inflammatory response (13); furthermore, initial low serum concentrations of Apo-A1 are poor prognostic indicators during severe sepsis, being associated with increased mortality, prolonged stay in intensive care unit, and hospital-acquired infection (14). Plasma lipoproteins concentrations might not simply reflect the severity of the disease, but they could directly modulate the host response to inflammation. Indeed, Apo-A1 can directly inactivate bacterial endotoxin, but also inhibits LPS-induced production of inflammatory cytokines from monocytes (15). Data regarding Apo-A1 concentrations and inflammation in dogs are scarce. According to a previous canine study (5), it could be hypothesized that Apo-A1 behaves as a negative acute phase protein in this species, based on the finding of a significant negative correlation between Apo-A1 and positive acute phase proteins (ferritin and CRP). In the present study, dogs with sepsis showed increased serum CRP concentrations and, at the same time, significantly lower serum Apo-A1 concentrations compared to a group of healthy dogs. However, a significant negative correlation between serum values of Apo-A1 and CRP concentrations was not found in our population. The low number of observations might be responsible for the latter finding; moreover, different kinetics or magnitude of the response or, as reported for people, different mechanisms other than systemic inflammation could play a role in the reduction in Apo-A1 during sepsis, including consumption by binding bacterial substances. As previously stated in people, the circulating level of SAA raises during infection, and SAA itself can displace Apo-A1 and becomes tightly associated with HDL (16). A parallel evaluation of serum SAA and Apo-A1 concentrations was not possible in this population of septic dogs, but further assessment in future studies on canine sepsis is warranted. By gathering septic dogs according to the most prevalent diseases in our population, we observed that mean serum albumin and CRP concentrations, and APPLE_fast_ score values were not significantly different among the subgroups, assuming a comparable condition in terms of intensity of systemic inflammation, protein loss, or disease severity. However, dogs with septic peritonitis showed significantly lower Apo-A1 concentrations compared to healthy dogs and to the ones with pyometra. Furthermore, it should be pointed out that dogs with septic peritonitis in our study had the highest frequencies of death compared to the other sepsis subgroups. If these preliminary findings might support the role of Apo-A1 as marker of disease severity in a specific setting of critical abdominal illness, like septic peritonitis, due to the low number of dogs included in the latter subgroup of sepsis, they need to be confirmed in a wider population. In this regard, when the overall septic population was considered, non-surviving dogs had significantly lower serum Apo-A1 concentrations and higher APPLE_fast_ scores compared to ones who survived. The present findings confirm the prognostic performance of the APPLE_fast_ score in canine SIRS (17), and further support a potential prognostic significance of Apo-A1 in septic dogs. However, the large overlap of Apo-A1 values among healthy dogs and most of the septic subgroups limited its diagnostic accuracy in this population, being only fair, based on the area under the ROC curve. Interestingly, in dogs who survived to hospital discharge, baseline values of serum Apo-A1 had a significant negative correlation with the days of hospitalization, assuming a potential role of Apo-A1 as a marker of disease severity. However, the strength of the relationship was weak, and the real biological significance is not relevant, thus far.

There are some limitations to be considered when interpreting the present results. Diagnosis of sepsis was based on the clinical SIRS criteria associated with a documented infection, but the retrospective nature of the study limited the data collection and the stratification of the dogs based on sepsis severity (e.g., presence of shock, need of vasopressors, presence of organ dysfunction). The evaluation of the APPLE_fast_ score, however, allowed us to partially overcome such limitation. Moreover, the small sample sizes of the identified sepsis subgroups might have limited the statistical power of the comparative analysis. Finally, healthy control dogs were not matched for age and breed variation with the population of septic dogs.

## Conclusion

Apo-A1 seems to behave as a negative acute phase protein and might support a clinical suspicion of sepsis in specific settings of critical illness. A potential diagnostic and prognostic significance of Apo-A1 in dogs with septic peritonitis is warranted by the present findings. However, further prospective studies focusing on its diagnostic and prognostic performance in selected septic diseases are needed.

## Data Availability Statement

The datasets generated for this study are available on request to the corresponding author.

## Ethics Statement

This animal study was reviewed and approved by the Animal Welfare Committee (COBA) of the AlmaMater Studiorum—University of Bologna (Bologna DL 26/2014, Project 846). Written informed consent was obtained from the owners for the participation of their animals in this study.

## Author's Note

Troìa, R. (2018) Biomarkers of sepsis and multiorgan dysfunction syndrome in critically ill dogs and cats [dissertation/PhD thesis]. [Bologna, (Italy)]: *Alma Mater Studiorum*—Università di Bologna. The preliminary results of this manuscript were presented as an oral presentation at the 17th EVECCS Congress, June 21th−23th 2018, Venice, Italy.

## Author Contributions

MG designed the study, analyzed data, co-wrote, and edited the manuscript. GG and RT co-wrote and edited the manuscript. FF and FD edited the manuscript. All authors contributed to read and approved the final manuscript.

## Conflict of Interest

The authors declare that the research was conducted in the absence of any commercial or financial relationships that could be construed as a potential conflict of interest.

## Abbreviations

Apo-A1: apolipoprotein A1
APPLE_fast_: acute patient physiologic and laboratory evaluation fast score
CRP: C-reactive protein
HDL: high density lipoprotein
LPS: lipopolysaccharide
ROC: receiver operating characteristic
SAA: serum amyloid A
SIRS: systemic inflammatory response syndrome.
